# UvKmt2-Mediated H3K4 Trimethylation Is Required for Pathogenicity and Stress Response in *Ustilaginoidea virens*

**DOI:** 10.3390/jof8060553

**Published:** 2022-05-24

**Authors:** Shuai Meng, Huanbin Shi, Chuyu Lin, Zhongling Wu, Fucheng Lin, Zeng Tao, Yanjun Kou

**Affiliations:** 1 State Key Lab of Rice Biology, China National Rice Research Institute, Hangzhou 311400, China; mengrice@163.com (S.M.); shihuanbin@caas.cn (H.S.); 2 State Key Lab of Rice Biology, Ministry of Agriculture Key Laboratory of Molecular Biology of Crop Pathogens and Insects, Institute of Biotechnology, Zhejiang University, Hangzhou 310058, China; 22016208@zju.edu.cn (C.L.); wzl_1995@163.com (Z.W.); fuchenglin@zju.edu.cn (F.L.); 3 State Key Laboratory for Managing Biotic and Chemical Threats to the Quality and Safety of Agro-Products, Institute of Plant Protection and Microbiology, Zhejiang Academy of Agricultural Sciences, Hangzhou 310021, China

**Keywords:** *false smut*, conidiation, virulence, Kmt2, H3K4me3, rice

## Abstract

Epigenetic modification is important for cellular functions. Trimethylation of histone H3 lysine 4 (H3K4me3), which associates with transcriptional activation, is one of the important epigenetic modifications. In this study, the biological functions of UvKmt2-mediated H3K4me3 modification were characterized in *Ustilaginoidea virens*, which is the causal agent of the false smut disease, one of the most destructive diseases in rice. Phenotypic analyses of the Δ*Uvkmt2* mutant revealed that *UvKMT2* is necessary for growth, conidiation, secondary spore formation, and virulence in *U. virens*. Immunoblotting and chromatin immunoprecipitation assay followed by sequencing (ChIP-seq) showed that *UvKMT2* is required for the establishment of H3K4me3, which covers 1729 genes of the genome in *U. virens*. Further RNA-seq analysis demonstrated that UvKmt2-mediated H3K4me3 acts as an important role in transcriptional activation. In particular, H3K4me3 modification involves in the transcriptional regulation of conidiation-related and pathogenic genes, including two important mitogen-activated protein kinases *UvHOG1* and *UvPMK1*. The down-regulation of *UvHOG1* and *UvPMK1* genes may be one of the main reasons for the reduced pathogenicity and stresses adaptability of the ∆*Uvkmt2* mutant. Overall, H3K4me3, established by histone methyltransferase *UvKMT2*, contributes to fungal development, secondary spore formation, virulence, and various stress responses through transcriptional regulation in *U. virens*.

## 1. Introduction

Epigenetic regulation depends on the modification of genomic DNA or histone without changing DNA sequences [1]. It is well known that the lysine methylation of histone H3 plays a vital role in epigenetic regulation. Among these histone H3 modifications, H3K4me3, which is trimethylation at the fourth lysine of histone H3 catalyzed by histone methyltransferase (HMT), is significantly enriched in genes with active transcription [2,3]. In *Saccharomyces cerevisiae*, H3K4me3 modification depends on the COMPASS (complex of proteins associated with SET1) composed of SET1/KMT2 and other proteins [4,5,6]. Studies on the functions of COMPASS revealed that H3K4me3 modification is required for reducing collisions between replication and transcriptional machinery to protect genome integrity and maintaining normal development in yeast [7,8,9]. *SET1*, trithorax (Trx), and trithorax-related (Trr) homologs of yeast H3K4 methyltransferase in *Drosophila* are required for controlling germline stem cells maintenance and germ cell differentiation [10,11]. In *Arabidopsis*, Set1-mediated H3K4 methylation plays a vital role in the transcriptional activation of *FLC* (*FLOWERING LOCUS C*), which is one of the major floral repressors and determinants for vernalization requirement [12,13]. Consistent with the universal association of H3K4 methylation with transcription, the SET1/MLL complex is associated with active transcription and plays important roles in a very wide range of development and physiology in mammals [14,15].

Trimethylation of H3K4 on chromatin also plays important roles in the growth, differentiations, and pathogenicity in filamentous fungi. In the model filamentous fungus *Neurospora crassa*, Set1/COMPASS histone H3 methyltransferase is involved in the regulation of circadian rhythm [16]. In another model, filamentous fungus *Aspergillus nidulans*, Set1 has also been shown to regulate mitosis as in yeast [6,17]. In the rice blast fungus *Magnaporthe oryzae*, disruption of Set1/Kmt2-mediated H3K4me3 modification results in defects of vegetative growth, appressorium formation, and pathogenicity [18]. In the rice bakanae pathogen *Fusarium fujikuroi*, Set1 is involved in secondary metabolism and conidiation [19]. Similarly, in the causal agent of maize ear rot *Fusarium verticillioides*, the wheat head blight fungus *Fusarium graminearum*, and *Aspergillus flavus*, deletion of Set1 leads to slow growth, blocked synthesis of some secondary metabolisms, and reduced virulence [20,21,22]. In the filamentous insect pathogenic fungus *Beauveria bassiana*, the H3K4me1/me2/me3 were abolished by inactivation of histone lysine methyltransferase SET1/KMT2, resulting in toxicity loss, reduced hydrophobicity of conidia, and increased sensitivities to cell wall stress [23]. Taken together, these studies indicate that the roles of H3K4me3 in fungi are conservative and diverse, which still needs to be further revealed.

*Ustilaginoidea virens* is one of the most devastating rice pathogens worldwide [24]. *U. virens* infection not only reduces the yield and quality of rice but also pollutes rice seeds with mycotoxins [25,26,27]. In recent years, the research on the infection process of *U. virens* has made great progress. During the rice booting stage, *U. virens* infects rice spikelets and fills floral organs and eventually forms false smut balls, which is the obvious and typical symptom of rice false smut disease [28]. The infection of *U. virens* blocks the nutrition transportation and normal development of grains, resulting in the increase in empty chaff rate and the decrease in grain weight [29,30]. In order to complete the infection, *U. virens* needs to coordinate acquiring nutrients from rice cells, eluding the plant’s immunity and adapting stresses from the host and the environment through different pathogenic mechanisms [31,32,33,34,35,36]. Revealing the underlying pathogenic mechanism of *U. virens* provides clues for developing more effective control strategies [35,36,37,38].

In this study, we found that the H3K4me-specific SET1/KMT2 is required for transcriptional activation of genes in *U. virens*. UvKmt2 localizes in the nuclei and is necessary for growth, conidiation, stress response, and virulence. Furthermore, combined with ChIP-seq and RNA-seq analyses of the ∆*Uvkmt2* mutant, we found that H3K4me3-mediated transcriptional activation was closely related to sporulation and pathogenic genes in *U. virens*.

## 2. Materials and Methods

### 2.1. Sequence Analysis

The sequences of the genes and proteins used in this study were downloaded from the National Center for Biotechnology Information (NCBI, https://www.ncbi.nlm.nih.gov/, accessed on 18 October 2021). The motifs of Kmt2 homologs were predicted by SMART (http://smart.embl-heidelberg.de/, accessed on 19 October 2021), and the phylogenetic analysis was performed using MEGA 7.0 with a neighbor-joining algorithm [39].

### 2.2. Strains and Growth Condition

The *U. virens* WT strain HWD-2 presented by Prof. Junbin Huang of Huazhong Agriculture University (China) was used for this study. The WT and all transformed strains derived from the WT were cultured on PSA (potato sucrose agar, potato 200 g/L, sucrose 20 g/L and agar 20 g/L) plates at 28 °C under dark conditions. To evaluate the vegetative growth, the mycelial plugs were inoculated on PSA plates for 14 d. In order to collect mycelia and conidia, the mycelial plugs were grown in liquid PS (potato sucrose, potato 200 g/L, sucrose 20 g/L) medium at 180 rpm for 7 d.

### 2.3. Conidial Germination Assay

For conidial germination, 5 μL of conidial droplets (1 × 10^6^ conidia/mL) was inoculated on water agar plates (Agarose 1.5 g/L,) and incubated at 28 °C for 3 d. Images were taken under an Olympus BX53 microscope equipped with bright field optics.

### 2.4. Vectors Construction and Transformation

To create the deletion mutants of *UvKMT2*, a gene replacement strategy was used in this study [35]. Briefly, approximately 1 Kb of 5′ UTR and 3′ UTR regions of *UvKMT2* were amplified from the genomic DNA of the WT strain and ligated sequentially to the flanking of *hygromycin resistance gene cassette* in the *pFGL821* (Addgene, 58223, Watertown, MA02472, USA). The resultant plasmid *pFGL821-UvKMT2* was introduced into the WT strain by *Agrobacterium tumefaciens*-mediated transformation (ATMT). The correct transformants were verified by PCR, qRT-PCR, and Southern blot assay (primers listed in Table S1) [35].

To generate the complementary vector, the *UvKMT2* fragment containing 2 Kb of promoter and coding region was amplified by *UvKMT2-cF*/*R* and ligated into the vector *pFGL823* [35]. To generate the localization vector, the same *UvKMT2* fragment was cloned to *pFGL820-GFP-TrpC* terminator [40]. After sequencing, the resultant plasmids were introduced into the Δ*Uvkmt2* by ATMT to obtain the complemented and GFP-tagging strains. All the correct transformants were confirmed by PCR and qRT-PCR assays (all primers listed in Table S1).

### 2.5. Inoculation Assay

The *U. virens* inoculation assay was performed as described [35]. Briefly, the WT, Δ*Uvkmt2*, and complemented strain Δ*Uvkmt2*-C were cultured in PS medium for 7 d. Then, the mycelia were broken into pieces in a juice blender to make a mixed suspension of mycelia and conidia. The conidial concentration of the mixture was adjusted to 1 × 10^6^/mL and then 2 mL of the mixture was injected into panicles of Wanxian 98 (a susceptible rice cultivar, *Oryza sativa* L. *indica*). After inoculation, the rice plants were cultivated at 22 °C with 95% humidity for 2 d following 28 °C for 3 weeks. The disease symptoms were shown by the images of infected panicles and the number of false smut balls. These experiments were repeated three times with more than 30 inoculated panicles each time. The values were represented as the mean ± SD from three independent replicates.

### 2.6. Subcellular Localization Analysis

The mycelia cultured in liquid PS were stained with 100 μg/mL Hoechst 33342 (Sigma, 14533, St. Louis, MO, USA) for 20 min to observe nuclei. LSM700 (Carl Zeiss Inc., Oberkochen, Germany) was used for epifluorescence microscopy imaging according to the conditions for detecting GFP or Hoechst signals. Image was processed using Fiji (http://fiji.sc/wiki/index.php/Fiji, accessed on 12 January 2021). 

### 2.7. Immunoblot Assay

To detect histone modification, 0.2 g of PS-cultured mycelia were ground in liquid nitrogen and suspended with buffer I (2 mM MgCl_2_, 20 mM KCl, 20 mM Tris pH 7.5, 250 mM Sucrose, 25% Glycerol, and 5 mM beta-mercaptoethanol) with 0.1 mM PMSF (phenylmethanesulfonyl fluoride) and 1 × proteinase inhibitors (Protease Inhibitor Cocktail, Roche, Basel, BS, Switzerland). The resulting mixture was filtered through one layer of Miracloth (Millipore, Burlington MA, USA) and centrifuged. Then, the buffer II (150 mM NaCl, 1 mM EDTA, 50 mM Tris-HCl pH 7.4, and 1% Triton100) with 1 × protein inhibitor was used to suspend the total nucleus proteins. Total nucleus proteins were separated by a 15% SDS polyacrylamide gel electrophoresis (SDS-PAGE) and transferred to PVDF membrane, subsequently detected by immunoblotting with anti-H3 (Huabio, M1309-1, Hangzhou, China), anti-H3K4me3 (Abcam, ab1012, Cambridge, UK), anti-H3K27me3 (Active motif, 39155, Carlsbad, CA, USA), or anti-H3K36me3 (Abcam, ab9050, Cambridge, UK) antibody, respectively. The immunoblots were captured with an imaging system using a chemiluminescence kit (Bio-Rad, Hercules, CA, USA).

### 2.8. Chromatin Immunoprecipitation (ChIP) and Sequencing

The ChIP assays were conducted as previously described [41,42]. Briefly, 7-d PS-cultured mycelia were cross-linked with 1% formaldehyde for 20 min. After the cross-linking was stopped with 125 mM glycine, the mycelia were ground in liquid nitrogen and suspended with the nuclei isolating buffer (10 mM Tris pH 8.0, 10 mM sodium butyrate, 400 mM sucrose, 0.1 mM PMSF, 5 mM β-mercaptoethanol, and 1 × proteinase inhibitors). The precipitated nuclei were treated with 1 mL lysis buffer (50 mM HEPES pH 7.5, 150 mM NaCl, 1mM EDTA, 10 mM sodium butyrate, 0.1% deoxycholate, 1% Triton X-100, 0.1% SDS, 1mM PMSF, and 1 × Roche protease inhibitor cocktail) and broken into DNA fragments between 200–500 bp using Diagenode Bioruptor. After pretreating with 10 μL of protein A beads (Thermo Fisher, 10001D, Waltham, MA, USA), the supernatant was incubated with anti-H3K4me3 antibody (Abcam, ab1012, Cambridge, UK) for 1 h. Then, 20 μL of protein A beads were added into the above reaction system to bind anti-H3K4me3 antibody. After washing three times, the DNA was recovered and used for ChIP-seq and ChIP-qPCR assays. Two biological repeats were conducted.

For ChIP-seq assay, the DNA recovered from ChIP assays was used to construct the library with the NEBNext Ultra II DNA Library Prep Kit for Illumina (NEB, E7645L, Ipswich, MA 01938). High-throughput sequencing of the library was carried out using Illumina Hiseq-PE150 by Novogene Corporation (Beijing, China). Subsequently, the clean paired-end reads were mapped to the reference genome with Bowtie2 (Version 2.3.5) and reads with low mapping quality or multiple positions on the genome were identified and removed by SAMtools (Version 1.9) [43,44]. Enriched peaks of perfectly and uniquely mapped reads were called and annotated by HOMER (Version 4.9.1) with default parameters [45]. The common peaks found in two biological replicates were converted to bigwig files using bamCoverage program in deepTools, which were imported into the Integrative Genomics Viewer (IGV, Broad institute and the Regents of the University of California) for visualization [46,47]. To assign peaks to proximal genes, the distance of 3.0 Kb flanking the peak summit was extracted. Then, the signal density was normalized and calculated within 3.0 Kb flanking of TSSs of coding and non-coding targets. The mean H3K4me3 levels at defined loci between WT and the Δ*Uvkmt2* mutant were compared using the computeMatrix, plotProfile, and plotHeatmap programs in deepTools [48].

To validate ChIP-seq results, the levels of examined fragments were relative to internal reference gene *UvACTIN* (*Uv8b_6104*) using quantitative real-time PCR. The PCR primers are listed in Table S1. Two biological repeats were conducted.

### 2.9. qRT-PCR (Quantitative Real-Time PCR) and RNA Sequencing

Total RNA was isolated from mycelia cultured in liquid PS for 7 d using the TRIzol (Invitrogen) reagent. Subsequently, cDNAs were synthesized with a reversely transcribed kit (TAKARA). The cDNA was subjected to qRT-PCR assay with SYBR Green qPCR Master Mix (TAKARA) with the *UvACTIN* gene (*Uv8b_6104*) as internal control (all primers listed in Table S1). All experiments were performed with three independent biological replicates.

RNAs were sequenced with Illumina Hiseq X-Ten with Hiseq-PE150 strategy by Novogene Corporation (Beijing, China). The obtained reads were mapped to the *Ustilaginoidea virens* (UV-8b) genome using Hisat2 (version 2.1.0) with default settings and sorted by SAMtools (Version 1.9) [49]. R v4.0.3 package DESeq2 (version 1.30.1) was used to identify genes that were differentially expressed between the WT and ∆*Uvkmt2* mutant (listed in Supplementary Table S2). Genes with at least a 2-fold change in expression level (log_2_Fold Change ≥ 1, *p* < 0.05) between the ∆*Uvkmt2* mutant and WT were considered to be differentially expressed. Gene ontology (GO) analysis for enriched biological processes, molecular function, and cellular component was performed on the website (https://www.omicshare.com/tools/, accessed on 6 November 2021). Cufflinks was used to quantify gene expression values as reads per kilobase per million mapped reads (RPKM), and cuffdiff was used to identify differentially expressed genes between WT and the ∆*Uvkmt2* strains [50].

Plot and Venn diagrams of overlap for the up- or down-regulated genes and H3K4me3 enrich genes were generated using GraphPad Prism 8 and webtool eVenn (http://www.ehbio.com/test/venn/#/, accessed on 7 November 2021) [51]. Scatter plot was generated from ggplot2 program (R package version 3.3.3, Robert Gentleman and Ross Ihaka, Auckland, AKL, New Zealand) in R (v4.0.3). To determine the significance of overlap in Venn diagrams, statistical testing of the overlap between two gene lists was performed on the website (http://nemates.org/MA/progs/overlap_stats.html, accessed on 7 November 2021). In the statistical testing of the overlap, the total number of genes in the *U. virens* genome used was 8297, which was obtained from the NCBI.

### 2.10. Stresses Treatments

To determine sensitivities to various stresses, vegetative growth of the WT, ∆*Uvkmt2* mutants, and complementation strains were observed after being grown on the PSA plates with 0.3 M NaCl, 0.5 M sorbitol, 0.03% SDS, 120 mg/mL CR, 120 mg/mL CFW, or 0.015% H_2_O_2_ for 14 d at 28 °C. Images were taken to show the vegetative growth under various stress conditions. The formula of relative inhibition rate was calculated as follow: growth inhibition rate = (diameters of strain colony on the PSA minus diameters of strain colony on the PSA amended with different chemicals)/diameters of the strain colony on the PSA × 100%. The values were represented as the mean ± SD from three independent replicates.

### 2.11. Data Availability

ChIP-seq and RNA-seq datasets generated in this study have been deposited in the Gene Expression Omnibus (GEO) repository under accession codes GSE203326 and GSE203327, respectively.

## 3. Results

### 3.1. Identification of UvKMT2 in U. virens

**Figure 1:**
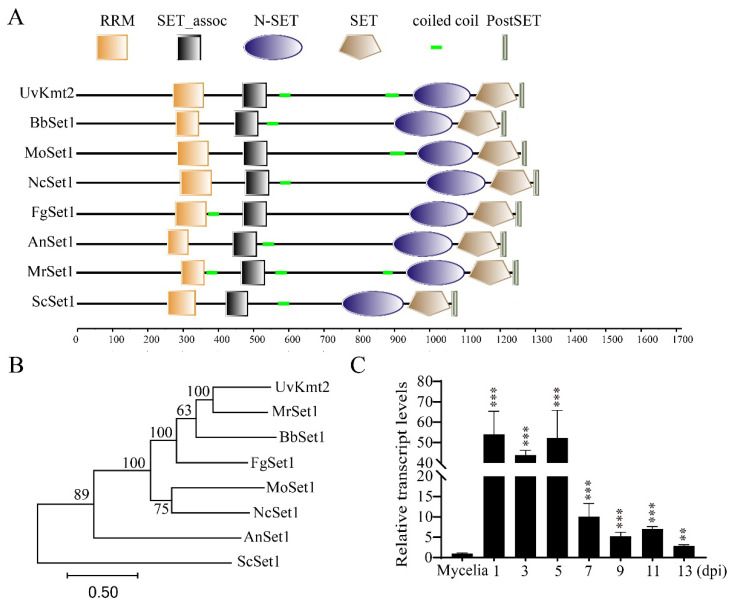
Identification of UvKMT2 in *U. virens*. (**A**) Functional domain analysis of Kmt2 homologs. The Kmt2 homologs include *Ustilaginoidea virens* Kmt2 (XP_042996622.1), *Beauveria bassiana* Kmt2 (XP_008600524.1), *Magnaporthe oryzae* Set1 (XP_003715029.1), *Neurospora crassa* Set1 (XP_961572.3), *Fusarium graminearum* Set1 (XP_011327217.1), *Aspergillus nidulans* Set1 (XP_663399.1), *Metarhizium robertsii* Set1 (XP_007823015.2), and *Saccharomyces cerevisiae* Set1 (NP_011987.1) were analyzed. (**B**) Phylogenetic tree of Kmt2 homologs was constructed using MEGA 7.0 with the neighbor-joining algorithm. The numbers close to the branch nodes are bootstrap values. (**C**) Expression pattern of *UvKMT2* was determined by qRT-PCR assay. The expression level of *UvKMT2* during infection process was calibrated to that of mycelia. The *β-actin* gene served as the internal control. The data represent the mean ± SD from three biological replicates. ***, *p* < 0.001; **, *p* < 0.005.

By BLASTp with *Neurospora crassa* Set1 (XP_961572.3), the Kmt2 homologs, including *Ustilaginoidea virens* Kmt2 (XP_042996622.1), *Beauveria bassiana* Kmt2 (XP_008600524.1), *Magnaporthe oryzae* Set1 (XP_003715029.1), *Neurospora crassa* Set1 (XP_961572.3), *Fusarium graminearum* Set1 (XP_011327217.1), *Aspergillus nidulans* Set1 (XP_663399.1), *Metarhizium robertsii* Set1 (XP_007823015.2), and *Saccharomyces cerevisiae* Set1 (NP_011987.1) were hit. Functional domain analysis using the SMART tool (http://smart.embl-heidelberg.de, accessed on 19 October 2021) revealed that all these Kmt2 homologs contain five domains, including RRM, SET_assoc, N-SET, SET, and PostSET. Among these domains, the RRM domain is responsible for RNA recognition, and the SET domain possesses histone methyltransferase activity (Figure 1A). Furthermore, a phylogenetic tree of Kmt2 homologs constructed using the MEGA 7.0 software showed that UvKmt2 is most similar to the *M. robertsii* Kmt2 (Figure 1B). These results suggested that Kmt2 is highly conserved and may serve as a histone methyltransferase in *U. virens* as other organisms (Figure 1B).

To gain insights into the possible function of *UvKMT2*, the expression level of *UvKMT2* was determined using qRT-PCR (quantitative real-time polymerase chain reaction) assay during *U. virens* infection stages. The results showed that, compared with that of the mycelial stage, the expression level of *UvKMT2* increased more than fiftyfold at 1, 3, and 5 dpi (days post inoculation) and more than fourfold at 7, 9, 11, and 13 dpi (Figure 1C). The transcriptional up-regulation of *UvKMT2* post inoculation implicated a possible role of *UvKMT2* during pathogenesis in *U. virens*.

### 3.2. Disruption and Complementation of UvKMT2

**Figure 2:**
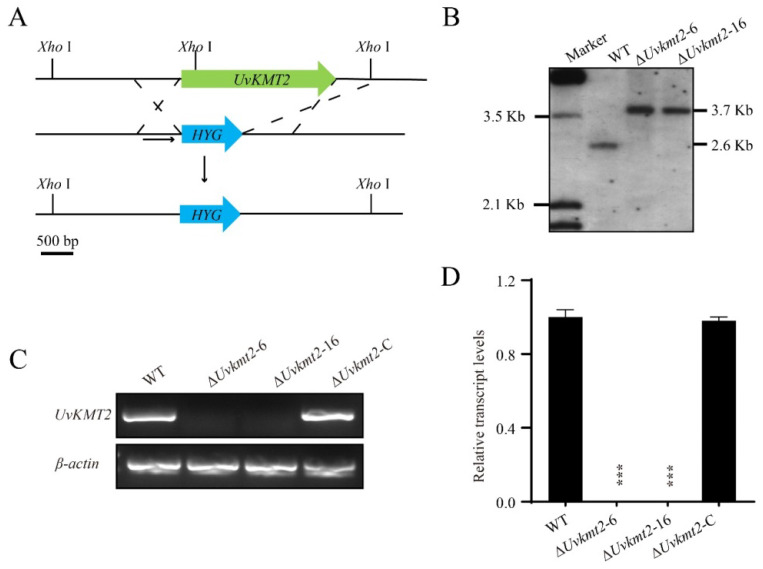
Disruption of *UvKMT2* and complementation analysis. (**A**) Strategic map of *UvKMT2* gene disruption and sites for restriction enzyme *Xho*I. *HYG*, *hygromycin resistance gene cassette*. Scale bar = 500 bp. (**B**) Southern blot analysis verified the correct *UvKMT2* deletion mutants. Genomic DNA of indicated strains was extracted and digested by *Xho*I. The probe for Southern blotting locates downstream of the *UvKMT2* coding region, as shown by the arrow line in (**A**). In the correct *UvKMT2* deletion mutants, a 2.6 Kb band in the HWD-2 was shifted to 3.7 Kb. Verification of *UvKMT2* deletion mutants and complementation strain was carried out by RT-PCR (**C**) and qRT-PCR (**D**) analyses. Obvious *UvKMT2* expression was detected in the WT and Δ*Uvkmt2*-C strains but not in the Δ*Uvkmt2*-6 and Δ*Uvkmt2*-16 mutants. Similar results were obtained from three biological repeats. Asterisks represent significant difference at *p* value < 0.001.

**Figure 3:**
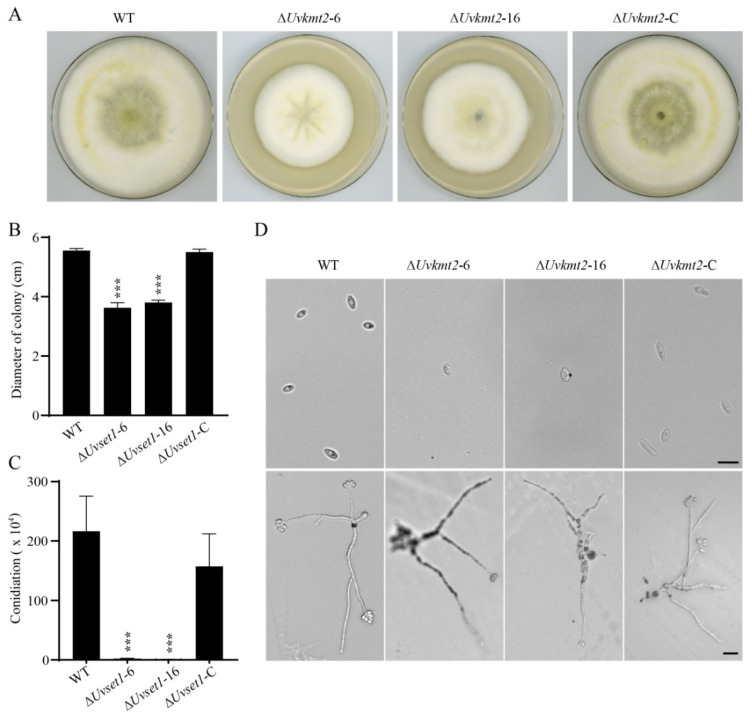
UvKmt2 is required for growth, conidiation, and formation of secondary spores in *U. virens*. (**A**) Colonies of the WT, Δ*Uvkmt2*, and complemented strains cultured on PSA at 28 °C in the dark. Photographs were taken at 14 d. (**B**) Statistical analysis of the colony diameters of indicated strains. (**C**) Knockout of *UvKMT2* gene led to highly decreased conidiation. The conidial concentration was determined after being cultured in PS medium for 7 d. Values represent the mean ± SD from three independent biological replicates. (**D**) Conidial germination of *U. virens* on water agar plates. A volume of 5 μL of conidial suspensions (1 × 10^6^ conidia/mL) was inoculated and incubated at 28 °C for 3 d. Scale bar, 5 μm. Error bars represent the standard deviations. *** indicates *p* value < 0.001 compared to the WT.

To reveal the biological functions of *UvKMT2* in *U. virens*, *UvKMT2* was deleted using a homologous recombination strategy along with the method of ATMT (*Agrobacterium tumefaciens*-mediated transformation) (Figure 2A). Subsequently, Southern blotting assay was used to verify the correct transformants, in which the *UvKMT2* locus was replaced by a *hygromycin resistance gene cassette* without an ectopic insertion. The results of Southern blotting assay showed that the 2.6 Kb band in the WT shifted to 3.7 Kb, indicating that Δ*Uvkmt2*-6 and -16 were correct deletion mutants (Figure 2B). In addition, RT-PCR and qRT-PCR results showed the target gene *UvKMT2* was only expressed in the WT, while not in the deletion mutants Δ*Uvkmt2*-6 and -16 (Figure 2C,D). Therefore, the Δ*Uvkmt2*-6 and -16 were chosen for further analyses. To confirm whether the altered phenotypes (Figure 3A) in the ∆*Uvkmt2* were caused by disruption of the *UvKMT2* gene, the WT copy of *UvKMT2* with its native promoter was reintroduced into the Δ*Uvkmt2*-16 mutant to generate the complementation strains. The expression level of the *UvKMT2* gene and the phenotypes of resultant Δ*Uvkmt2*-C strains were similar to those of the WT strain (Figure 2D and Figure 3A), indicating that *UvKMT2* functionally restores the defects in the ∆*Uvkmt2* mutant.

### 3.3. UvKMT2 Facilitates Growth, Conidiation, and Secondary Spore Formation

Since the colonies of ∆*Uvkmt2* mutants looked smaller than that of the WT strain, mycelial growth was measured by inoculating mycelial plugs of the WT, Δ*Uvkmt2*-6, -16, and Δ*Uvkmt2*-C on the PSA (potato sucrose agar medium) plates for 14 d. Compared with the WT, Δ*Uvkmt2*-6 and 16 were reduced in colony diameters (Figure 3A,B). In contrast, the mycelial growth defect of ∆*Uvkmt2* was rescued in the Δ*Uvkmt2*-C strain (Figure 3A,B). These results indicated that *UvKMT2* is required for the fungal growth in *U. virens*.

Conidia play an important role in the infection of *U. virens*. To investigate the function of *UvKMT2* in conidiation in *U. virens*, the same numbers of mycelial plugs of the WT, Δ*Uvkmt2*, and Δ*Uvkmt2*-C strains were cultured in the same volume of the liquid PS medium. After incubation at 28 °C with shaking for 7 d, conidia in the medium were measured and imaged under a microscope. The results showed that the Δ*Uvkmt2* mutants exhibited fewer conidia than those of the WT and complemented strain Δ*uvkmt2*-C (Figure 3C). The number of conidia was decreased by 90% in the Δ*uvkmt2* compared to the WT, but no morphological defects were observed in the conidia of Δ*uvkmt2*. These results suggested that *UvKMT2* is required for asexual development in *U. virens*.

During pathogenesis of *U. virens*, the formation of secondary spores tends to greatly increase the amount of inoculation that can be used to infect rice plants [28]. To investigate the conidial germination, conidia of the WT, Δ*uvkmt2*, and Δ*uvkmt2*-C strains were inoculated on the water agar plates and then cultured at 28 °C for 3 d. The result showed that there were no significant differences observed in the germination of conidia between the WT strain and ∆*Uvkmt2* mutant. However, the formation of secondary spores was highly reduced in the ∆*Uvkmt2* mutant comparing with those of the WT and complemented strains (Figure 3D), indicating that *UvKMT2* plays important roles in the formation of secondary spores in *U. virens*.

### 3.4. UvKMT2 Is Required for Virulence in U. virens

**Figure 4:**
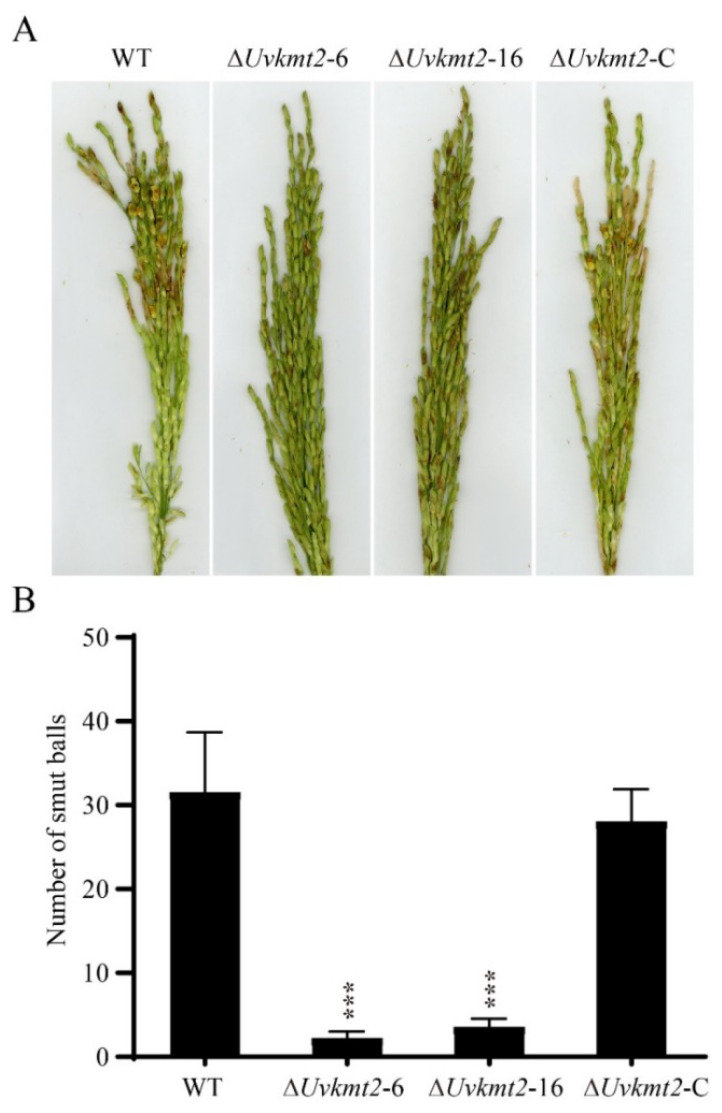
Deletion of *UvKMT2* gene resulted in reduced virulence. (**A**) Virulence assays of the WT, Δ*Uvkmt2*, and complemented strains. The conidia from indicated strains were injected into the panicles of rice plants (*Oryza sativa* L., cultivar Wanxian 98) at booting stage. The photos were taken at 21 dpi (days post inoculation). (**B**) The false smut balls inoculated with the Δ*Uvkmt2* strain were fewer than those of the WT and complemented strains. At least three independent biological experiments were performed with 30 inoculated panicles each time. Error bars represent the standard deviations. The data were subjected to Duncan’s test, and the significant differences were indicated by asterisks (***, *p* < 0.001).

The highly reduced formation of secondary spores may lead to the pathogenic defects in the ∆*Uvkmt2* mutant. To determine whether *UvKMT2* is required for the virulence of *U. virens*, the conidial suspensions of the WT, Δ*uvkmt2*-6 and -16, and complemented strains were injected into the booting-stage panicles of susceptible rice plants, Wanxian 98 (*Oryza sativa* L. indica), respectively. After 21 d of incubation, the Δ*Uvkmt2*-6 and -16 developed a few false smut balls (approximately five) on each inoculated panicle (Figure 4A,B). In contrast, approximately 30 diseased grains with false smut balls were found on each spike inoculated with the WT strain. Moreover, the reintroduction of *UvKMT2* restored the pathogenicity to the WT levels. These results indicated that *UvKMT2* plays an important role in the fungal virulence in *U. virens*.

### 3.5. UvKmt2 Is Essential for the Establishment of Histone Modification H3K4me3

**Figure 5:**
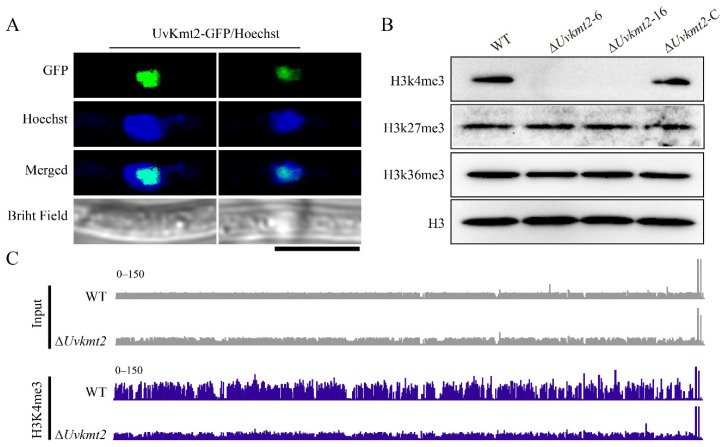
UvKmt2 is essential for establishing H3K4me3 modification. (**A**) Confocal microscopy-based subcellular localization of UvKmt2 fused with GFP. The GFP signal co-localized with Hoechst-stained nuclei. Scale bar = 5 μm. (**B**) Deletion of *UvKMT2* resulted in loss of H3K4me3 modification. Total nuclear proteins of WT, Δ*Uvkmt2*, and complemented strain Δ*Uvkmt2*-C were isolated to detect histone modification. Immunoblot assay were performed using H3, H3K4me3, H3K27me3, and H3K36me3 antibodies. (**C**) Genome browser views of H3K4me3 in the WT and ∆*Uvkmt2* mutant. H3K4me3-marked genomic sequences in the WT and Δ*Uvkmt2* were immunoprecipitated with H3K4me3 antibodies and then sequenced. The H3K4me3 enrichment was presented in the WT and Δ*Uvkmt2* strains. Similar results were obtained from two biological replicates.

To investigate whether UvKmt2 functions as histone methyltransferase, we first fused UvKmt2 with GFP and examined its subcellular localization in *U. virens*. As shown in the Figure 5A, *UvKMT2-GFP* were co-localized Hoechst-stained nuclei, suggesting that UvKmt2 localizes in the nucleus like Kmt2 homologs in other species. To further investigate whether *UvKMT2* is indeed responsible for the H3K4me3 modification, nucleic proteins of WT, Δ*Uvkmt2*-6 and -16, as well as the complemented strains were subjected to immunoblotting with a specific H3K4me3 antibody. The results showed that the H3K4me3 was detectable in the WT and complemented strains but nearly undetectable in the Δ*Uvkmt2*-6 and -16 mutants (Figure 5B). In contrast, the H3K27me3 and H3K36me3 levels showed no significant changes after deletion of the *UvKMT2* gene (Figure 5B). These results indicated that UvKmt2 is specifically required for H3K4me3 modification in *U. virens*.

To further depict whether H3K4me3 directly deposits on the chromatin, the genome-wide H3K4me3 occupancy was mapped using chromatin immunoprecipitation assay followed by sequencing (ChIP-seq) in the WT and Δ*Uvkmt2*-16 strains. The average enrichment of H3K4me3 in the WT strain was high and distributed in the specific chromosomal regions (Figure 5C and Table S2). In contrast, the H3K4me3 occupancy in the Δ*Uvkmt2*-16 strain was almost undetectable (Figure 5C), which was consistent with the aforementioned immunoblotting results with the H3K4me3 antibody. Compared with the Δ*Uvkmt2*-16 strain, 1749 significant peaks in total were examined in the WT strain (Log_2_Fold Change > 1, *p* < 0.05), which corresponded with 1729 genes. In animals, H3K4me3 modifications are mainly distributed in the downstream of the promoter and transcription start site [51,52]. In yeast, *Arabidopsis*, and rice, H3K4me3 modifications are concentrated in the promoter region downstream of the transcription start site [53,54,55]. In conclusion, all these results further suggested that UvKmt2 is essential for H3K4me3 modification in *U. virens*.

### 3.6. UvKmt2-Mediated H3K4me3 Plays a Critical Role in Transcriptional Activation

**Figure 6:**
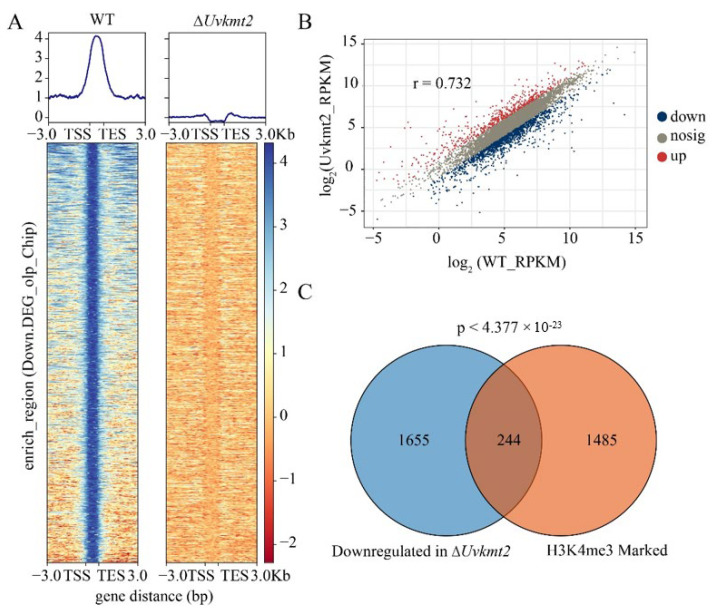
UvKmt2-mediated H3K4me3 plays a major role in transcriptional activation. (**A**) The average H3K4me3 occupancy within 3.0 Kb genomic regions flanking the summit of H3K4me3 peaks in the WT and ∆*Uvkmt2* strains. (**B**) Scatter plots reveal a positive correlation of up- and down-regulated genes between the Δ*Uvkmt2* and WT strains. Red dot, blue dot, and gray dot, respectively, represent an up-regulated gene, a down-regulated gene, and a gene without significant change. The RPKM for the Δ*Uvkmt2* was plotted against biological WT, demonstrating that three biological replicates are correlated. r is the correlation coefficient. (**C**) The overlapping between down-regulated genes in Δ*Uvkmt2* versus WT and H3K4me3-marked genes was presented by Venn diagrams.

Accumulating evidence established that Kmt2-mediated H3K4me3 modification plays important roles in transcriptional regulation [23]. In this study, we found that 1749 significant peaks are mainly distributed within gene bodies (Figure 6A). To investigate the roles of H3K4me3 in the transcriptional regulation in *U. virens*, RNA-seq analysis was conducted using PS (potato sucrose) cultured mycelia of WT and Δ*Uvkmt2*. The analysis of the reads per kilobase per million mapped reads (RPKM) from three biological replicates showed strong correlation between replicate experiments (Figure 6B). Compared with WT, a total of 3703 DEGs (differentially expressed genes) were obtained in the Δ*Uvkmt2* strain (*p* < 0.05), of which 1804 genes were up-regulated (log_2_ Fold Change > 1) and 1899 genes were down-regulated (log_2_ Fold Change < −1) (Tables S3 and S4). Gene ontology (GO) analysis showed that DEGs are involved in the various important biological processes, including cell development and differentiation, as well as response to external stimuli (Figure S1). These data indicated that the UvKmt2-mediated H3K4me3 is involved in a wide range of biological processes in *U. virens*.

By comparing the differentially expressed genes of RNA-seq and H3K4me3-occupied genes of ChIP-seq, we found that 244 of 1899 down-regulated genes in the Δ*Uvkmt2* strain were enriched by H3K4me3 modification in the WT (Figure 6C). These results suggested that the UvKmt2-mediated H3K4me3 plays a critical role in transcriptional expression in *U. virens*.

### 3.7. UvKmt2-Mediated H3K4me3 Modification Regulates Transcription of Conidiation Related and Pathogenic Genes

Aforementioned experimental results indicated that *UvKMT2* is required for conidiation and virulence. To comprehensively understand the transcriptional regulation of conidiation related and pathogenic genes by H3K4me3 modification, the expression levels of identified sporulation and pathogenic genes were compared in the WT and Δ*Uvkmt2* strains using the RNA-seq and ChIP-seq data. In contrast to the WT, at least 10 genes, whose homolog genes are involved in sporulation and pathogenesis in *M. oryzae* [36,38,49,56,57,58,59,60,61], were significantly down-regulated and absent of H3K4me3 modification in the Δ*Uvkmt2* strain (Figure S2A). These genes included *Uv8b_1008* (*Trehalose phosphate synthase*), *Uv8b_1325* (*MAP kinase kinase kinase Ste11*), *Uv8b_135* (*PHD transcription factor*), *Uv8b_2284* (*DUF1339 domain protein gene*), *Uv8b_3608* (*RhoGAP domain-containing protein gene*), *Uv8b_7958* (*Chitin synthase 1*), *Uv8b_363* (*Integral membrane protein gene*), *Uv8b_2084* (*Regulator of conidiation*), *Uv8b_2650* (*Dynamin GTPase effector*), and *Uv8b_2934* (*Phosphotransferase family gene*). To verify RNA-seq and ChIP-seq results of these genes, qRT-PCR and ChIP-qPCR analyses were carried out. Consistent with RNA-seq and ChIP-seq data, deletion of *UvKMT2* resulted in the down-regulation of these conidiation and pathogenicity-associated genes, which were related to the reduced H3K4me3 occupancy (Figure S2). It is possible that H3K4me3 modification is involved in conidiation and virulence by regulating the transcription of these 10 genes and other related genes.

**Figure 7:**
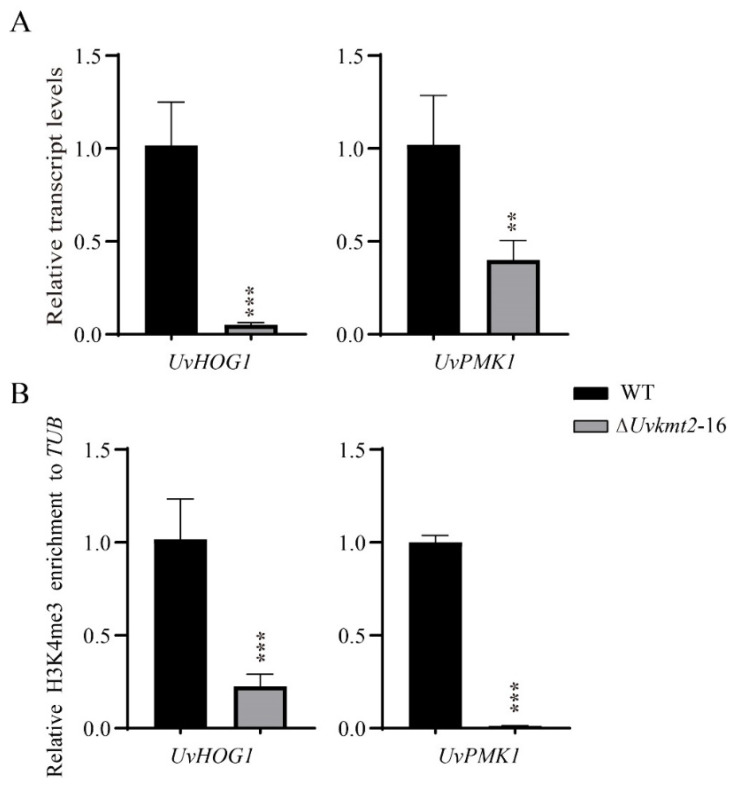
UvKmt2-mediated H3K4me3 modification regulates the transcription of *UvHOG1* and *UvPMK1* in *U. virens*. qRT-PCR (**A**) and ChIP-qPCR (**B**) assays of *UvHOG1* and *UvPMK1* genes. DNA immunoprecipitation with anti-H3K27me3 antibody was used as template to detect *UvHOG1* and *UvPMK1* genes enrichment in the WT and Δ*Uvkmt2* strains. Data represent mean ± SD of three independent biological replicates. Duncan’s test and the significant differences were indicated by asterisks (**, *p* < 0.005; ***, *p* < 0.001).

In addition, two important mitogen-activated protein kinase *UvHOG1* (*Uv8b_1888*) and *UvPMK1* (*Uv8b_2494*) genes were significantly down-regulated in the Δ*Uvkmt2* strain by comparing with the WT (Figure 7A). To verify these RNA-seq results, qRT-PCR assays were carried out. Consistently, the expression levels of *UvHOG1* and *UvPMK1* in the *UvKMT2* deletion mutant were 86% and 55% lower than those of the WT, respectively (Figure 7A). Furthermore, ChIP-qPCR assay showed that the decreased expression of *UvHOG1* and *UvPMK1* genes in the *UvKMT2* deletion is associated with reduced H3K4me3 occupancy (Figure 7B). These results suggested that UvKmt2 participates in the regulation of *UvHOG1* and *UvPMK1* expression by H3K4me3-mediated activation in *U. virens*. It was previously shown that *UvHOG1* and *UvPMK1* are involved in the pathogenicity of *U. virens*, and *UvHOG1* may also be necessary for virulence [36,38]. Therefore, one of the possible reasons for the reduced virulence in the ∆*Uvkmt2* mutant is that it was caused by the reduced H3K4me3 modification at the chromatin of *UvHOG1* and *UvPMK1* genes and the down-regulation of their expression.

### 3.8. UvKMT2 Is Involved in Various Stresses Adaption

**Figure 8:**
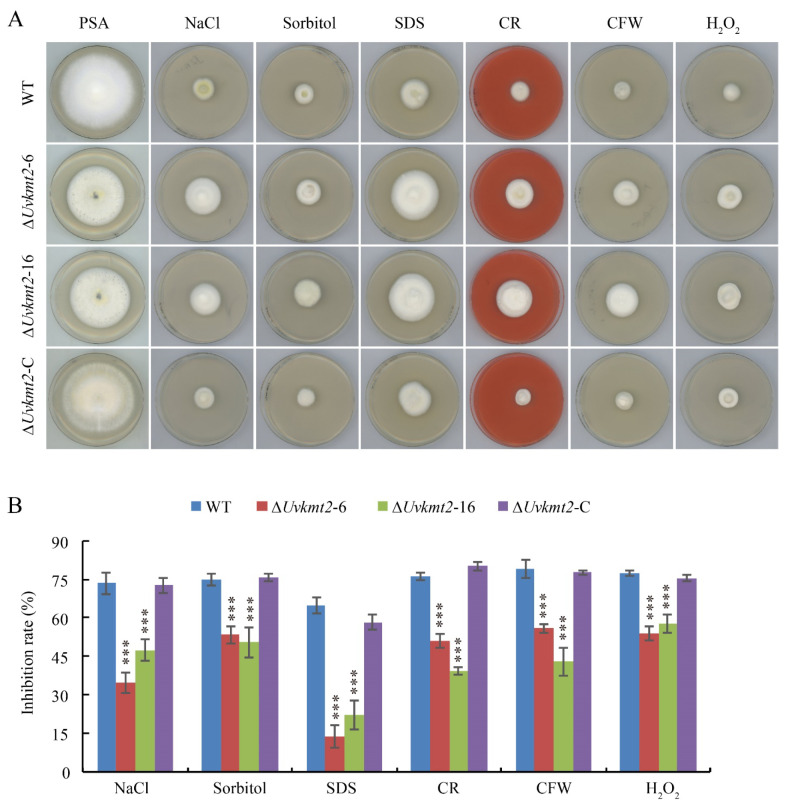
UvKmt2-mediated H3K4me3 modification is involved in various stresses adaption. (**A**) Colony morphology of indicated strain in the presence of different stress-mimicking chemicals. Mycelial plugs were inoculated on PSA plates supplemented with NaCl, sorbitol, SDS, Congo red, CFW, or H_2_O_2_ for 14 d. The Δ*Uvkmt2*-6 and -16 mutants were more sensitive to osmotic, cell wall, and oxidative stresses. (**B**) Statistic analysis of the relative growth inhibition rate under osmotic, cell wall, and oxidative stress conditions. Error bars represent the standard deviations from three independent replicates. ***, *p* < 0.001.

In addition to being critical to pathogenesis, *UvHOG1* and *UvPMK1* are also known to be involved in various stresses adaption [36,38]. The significantly decreased H3K4me3 occupancy and relative transcription level of *UvHOG1* and *UvPMK1* genes when *UvKMT2* was deleted suggest that stress responses in the ∆*Uvkmt2* mutant may be changed. Genes encoding the cell wall components, chitin synthase, and hyperosmotic responding proteins play an important role in stress response [30,36,38]. Based on RNA-seq analysis, we found that the hyperosmotic responding gene *UV8b_489*, the cell wall component encoding genes *UV8b_3186* and *UV8b_2857*, and the chitin synthase genes *UV8b_3637* and *UV8b_3908* were confirmed to be up-regulated by qRT-PCR assay (Figure S3). To test whether *UvKMT2* plays a role in stress adaptation, the WT, ∆*Uvkmt2*, and ∆*Uvkmt2*-C strains were cultured on the PSA and PSA amended with osmotic stress reagents NaCl and sorbitol, cell wall stress reagents SDS (sodium dodecyl sulfate), CFW (calcofluor white), and CR (Congo red) or oxidative stress reagent H_2_O_2_ for 14 d. Compared with the WT and ∆*Uvkmt2*-C strains, the colonies of Δ*Uvkmt2*-6 and -16 strains were smaller and exhibited high sensitivity to all tested stress-mimicking chemicals including NaCl, sorbitol, SDS, CFW, CR, and H_2_O_2_ (Figure 8). Therefore, UvKmt2 positively regulated the response to osmotic, cell wall, and oxidative stresses, possibly by regulating the expression of *UvHOG1* and *UvPMK1* genes, in *U. virens*.

## 4. Discussion

The epigenetic modification of H3K4me3 established by Kmt2 plays critical roles in organisms [23,62]. The filamentous fungus *U. virens* causes rice false smut disease, which is one of the most devastating rice fungal diseases in the rice-cultivated areas of the world [24]. In order to demonstrate the biological roles of KMT2-mediated H3K4me3 modification in *U. virens*, this study investigated the function of the methyltransferase Kmt2 homolog UvKmt2. It was found that *UvKMT2*-mediated H3K4me3 modification is required for vegetative growth and pathogenicity in *U. virens*. Furthermore, ChIP-seq assay and transcriptome profiling in combination with phenotypic analysis revealed that UvKmt2 participates in the transcriptional activation. In particular, UvKmt2 regulates the expression of the two important virulence genes *UvHOG1* and *UvPMK1*. The down-regulation of *UvHOG1* and *UvPMK1* genes may be one of the main reasons for the reduced pathogenicity and stresses adaptability in the ∆*Uvkmt2* mutant.

With the identification of pathogenic genes, the infection mechanisms of *U. virens* have been gradually revealed, but they remain unclear. In this study, an epigenetic regulator, UvKmt2, was demonstrated to be associated with the virulence of *U. virens*. First of all, deletion of *UvKMT2* resulted in the decreased conidiation, which usually leads to a reduction in the pathogenicity of *U. virens* [63]. For example, cAMP signaling pathway components UvAc1 and UvPdeH and transcriptional factors UvPro1, UvCom1, and UvHox2 involved in conidiation are required for the virulence [64,65]. Considering that H3K4me3 modification mediated by Kmt2 is involved in the genome-wide transcriptional regulation, the reduced conidiation in the ∆*Uvkmt2* mutant may be due to the expression level changes of conidiation-related genes. In addition, highly reduced formation of secondary spores may also be one of the reasons for reduced virulence in the ∆*Uvkmt2* mutant. In *U. virens*, the formation of secondary spores tends to greatly increase the amount of inoculation that can be used to infect rice plants [28]. Deletion mutants of the autophagy marker gene *UvATG8*, the putative plasma membrane phosphatase encoding gene *UvPSR1*, and general stress response gene *UvWHI2* were significantly reduced in pathogenicity due to the decrease in the production of secondary spores [35]. Moreover, studies have shown that the changes in various stresses adaption may also lead to reduced virulence. For instance, a lack of the cAMP signaling pathway components UvAc1 and UvPdeH or MAPK UvPmk1 showed differential sensitivity to stress-mimicking reagents and reduced pathogenicity [36,37]. In addition, the deletion of *UvKMT6* resulted in the alteration of stress adaption in *U. virens*, which has been implicated in linking to virulence [30]. Therefore, we thought that the stress response mediated by *UvKMT2* is also indispensable for virulence in *U. virens*. Taken together, *UvKMT2* contributes to the virulence of *U. virens* likely by regulating the hyphal growth, conidiation, formation of secondary spores, and ability of the stress response.

In this study, we found that *UvKMT2* appears to be a positive regulator in responding to osmotic, cell wall, and oxidative stresses in *U. virens*. In the *UvKMT2* deletion mutant, the expression levels of *UvHOG1* and *UvPMK1* were down-regulated, and H3K4me3 modification on these genes was removed. In *U. virens*, knockout of the MAPK encoding gene *UvHOG1* resulted in increased sensitivity to osmotic and cell wall stresses [38]. Therefore, the reduced expression of *UvHOG1* may be one of the explanations for the high sensitivity of Δ*Uvkmt2* mutants to osmotic, cell wall, and oxidative stresses. In addition, the knockout mutant of another MAPK encoding gene, *UvPMK1*, exhibited decreased tolerance to oxidative stress, which was consistent with the down-regulated expression of *UvPMK1* and increased sensitivity to oxidative stress in the Δ*Uvkmt2* mutant [36]. The underlying mechanism of *UvKMT2*-mediated H3K4me3 modification on the expression of stress-response-related genes needs to be further studied.

In conclusion, our results demonstrate the critical roles of *UvKMT2*-mediated H3K4me3 modification in transcriptional activation during growth, virulence, and stress responses in *U. virens*. These results extend the current understanding of the epigenetic modification of plant pathogenic fungi and provide novel and important insights into the pathogenic mechanisms of *U. virens*.

## Data Availability

The data presented in this study are available on request from the corresponding authors.

## Supplementary Materials

The following supporting information can be downloaded at: https://www.mdpi.com/article/10.3390/jof8060553/s1, Figure S1. GO enrichment analysis of differentially expressed genes between the WT and ∆*Uvkmt2* strains. The most enriched GO terms were categorized as biological processes, molecular function, and cellular component. Figure S2. UvKmt2-mediated H3K4me3 modification is required for the activation of sporulation and pathogenic genes in *U. virens*. (A) Heatmaps of the expression levels of 10 down-regulated genes putatively involved in sporulation and virulence in the Δ*Uvkmt2* mutant. (B) The relative transcriptional levels of representative sporulation and pathogenic-related genes were determined by qRT-PCR analysis. (C) ChIP-qPCR assay verified the enrichment of H3K4me3 modification on the chromatin of sporulation and pathogenic-related genes. DNA immunoprecipitation with anti-H3K4me3 antibody was used as template to detect relative enrichment in the WT and Δ*Uvkmt2* strains. Data represent mean ± SD of three independent biological replicates. *, ** or *** represent *p* value < 0.01, < 0.005, or < 0.001 compared with that of WT. Figure S3. qRT-PCR analysis of the expression levels of the genes related to cell wall, chitin synthase, and hyperosmotic stress between WT and ∆*Uvkmt2*. Data represent mean ± SD of three independent biological replicates. *** represent *p* value < 0.001 compared with that of WT. Table S1. Primers used in this study. Table S2. Genes marked by H3K4me3 modification. Table S3. Up-regulated genes in the ∆*Uvkmt2* mutant. Table S4. Down-regulated genes in the ∆*Uvkmt2* mutant.
