# Supplementary material for: UvKmt2-Mediated H3K4 Trimethylation Is Required for Pathogenicity and Stress Response in Ustilaginoidea virens
Source: J Fungi (Basel). 2022 May 24;8(6):553. doi: 10.3390/jof8060553 (PMC9225167; doi:10.3390/jof8060553)
Supplement: Supplementary file 1 [file jof-08-00553-s001.zip › jof-1714481-supplementary.pdf]

Supplementary Materials

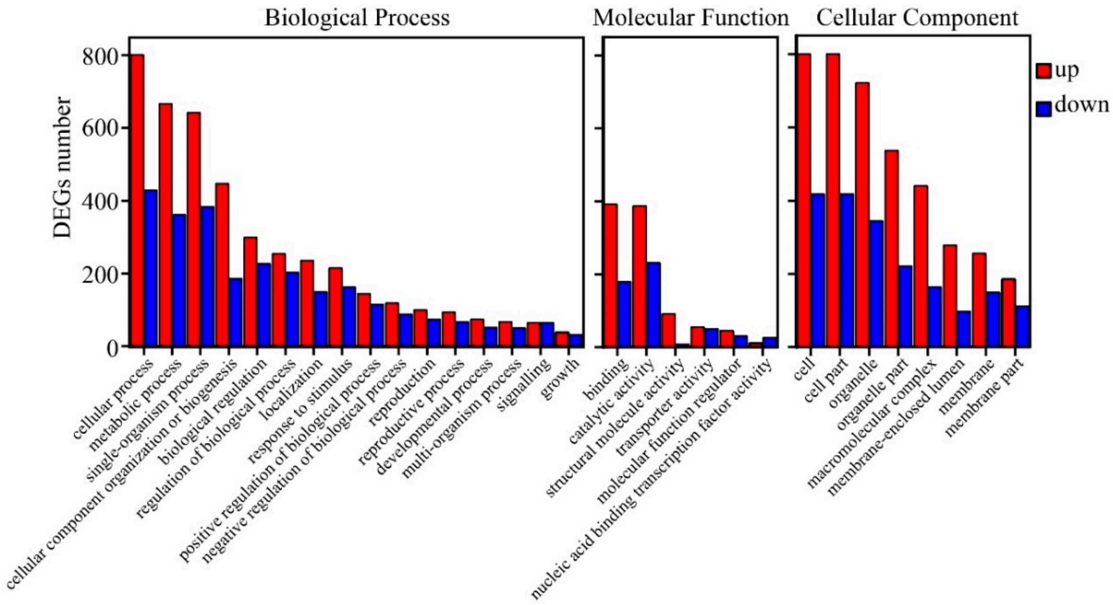

Figure S1. GO enrichment analysis of differentially expressed genes between the WT and Δ*Uvkmt2* strains. The most enriched GO terms were categorized as biological processes, molecular function, and cellular component.

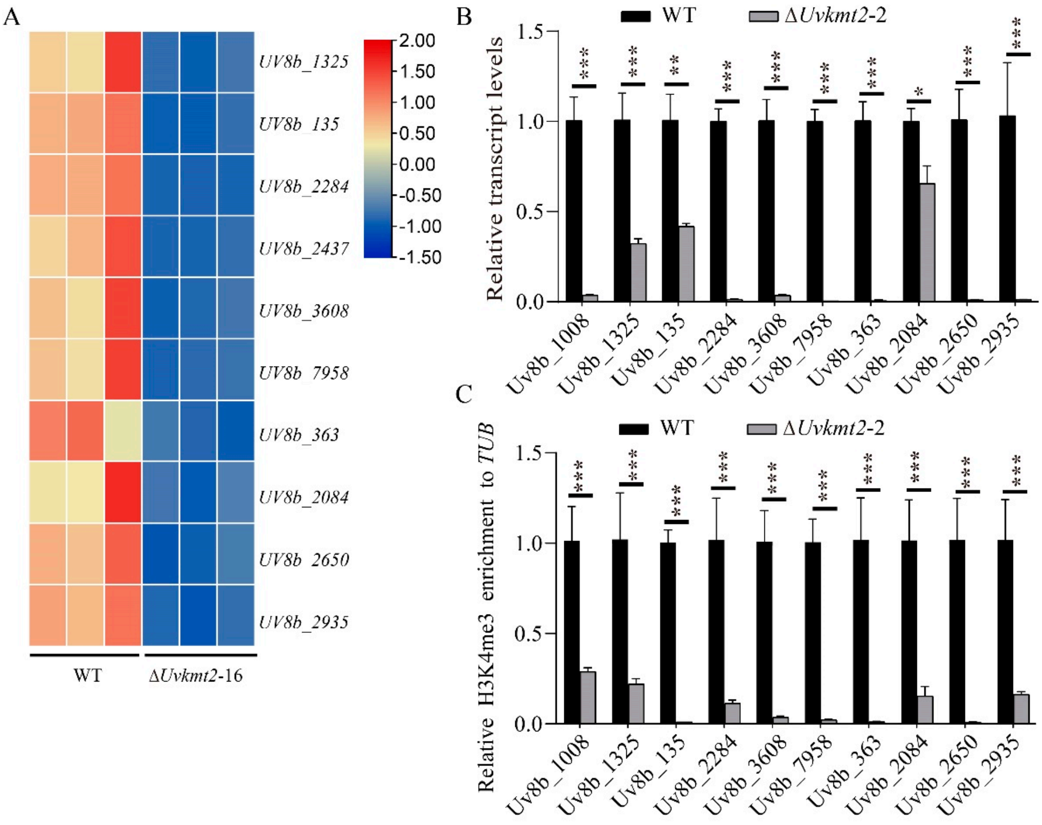

Figure S2. UvKmt2-mediated H3K4me3 modification is required for the activation of sporulation and pathogenic genes in *U. virens*. (A) Heatmaps of

the expression levels of 10 down-regulated genes putatively involved in sporulation and virulence in the Δ*Uvkmt2* mutant. (B) The relative transcriptional levels of representative sporulation and pathogenic-related genes were determined by qRT-PCR analysis. (C) ChIP-qPCR assay verified the enrichment of H3K4me3 modification on the chromatin of sporulation and pathogenic-related genes. DNA-immunoprecipated with anti-H3K4me3 antibody was used as template to detect relative enrichment in the WT and Δ*Uvkmt2* strains. Data represents mean ± SD of three independent biological replicates. *, ** or *** represent P value < 0.01, < 0.005 or < 0.001 compared with that of WT.

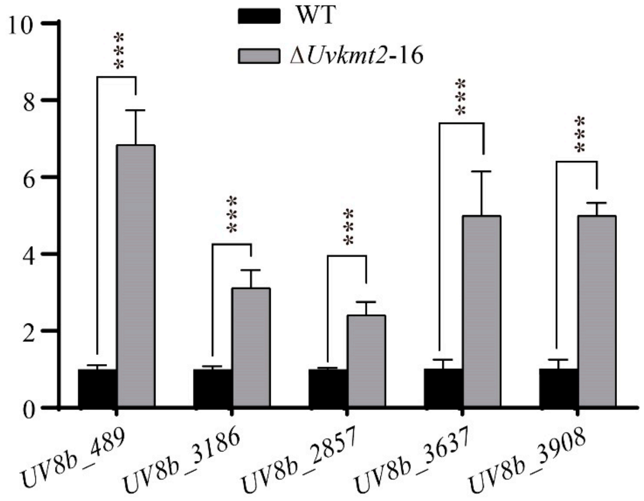

Figure S3. qRT-PCR analysis of the expression levels of the genes related to cell wall, chitin synthase, and hyperosmotic stress between WT and Δ*Uvkmt2*. Data represents mean ± SD of three independent biological replicates. *** represent P value < 0.001 compared with that of WT.

Table S1. Primers used in this study.

Table S2. Genes marked by H3K4me3 modification.

Table S3. Up-regulated genes in the Δ*Uvkmt2* mutant.

Table S4. Down-regulated genes in the Δ*Uvkmt2* mutant.
